# Effect of Gestational Weight Gain during the First Half of Pregnancy on the Incidence of GDM, Results from a Pregnant Cohort in Northern Greece

**DOI:** 10.3390/nu15040893

**Published:** 2023-02-10

**Authors:** Antigoni Tranidou, Emmanuela Magriplis, Ioannis Tsakiridis, Nikolaos Pazaras, Aikaterini Apostolopoulou, Michail Chourdakis, Themistoklis Dagklis

**Affiliations:** 1 3rd Department of Obstetrics and Gynaecology, School of Medicine, Faculty of Health Sciences, Aristotle University of Thessaloniki, 546 42 Thessaloniki, Greece; 2 Department of Food Science and Human Nutrition, Agricultural University of Athens, Iera Odos 75, 118 55 Athens, Greece; 3 Laboratory of Hygiene, Social & Preventive Medicine and Medical Statistics, School of Medicine, Faculty of Health Sciences, Aristotle University of Thessaloniki, 541 24 Thessaloniki, Greece

**Keywords:** gestational, diabetes, GDM, weight, gain, generalized linear model

## Abstract

The aim of this study was to evaluate the effect of gestational weight gain (GWG) up to 23^+6^ weeks of gestation on the incidence of Gestational Diabetes Mellitus (GDM). A pregnant cohort of 5948 women in Northern Greece was recruited. Anthropometric features before and during pregnancy were recorded, the GWG by 23^+6^ weeks was calculated and a Generalized Linear Regression Model (GLM) with subgroup analyses based on weight status were computed. GDM was diagnosed in 5.5% of women. GLM results showed that GDM likelihood increased with maternal age (MA) and pre-pregnancy BMI (aOR: 1.08, 95%CI: [1.06, 1.11] and aOR: 1.09, 95%CI: [1.09, 1.11], respectively). Ιn the normal pre-pregnancy weight group, when the extra weight gain was >8 kgs, the odds of GDM increased (OR: 2.13, 95%CI: [0.98, 4.21], *p* = 0.03). Women with pre-pregnancy level 2 clinical obesity (OB2 pre) (BMI > 35 and <40 kg/m^2^) that shifted to OB3 category (BMI ≥ 40 kg/m^2^) had an increased GDM likelihood (OR: 4.85, 95%CI: [1.50, 15.95]). Women of higher MA may require stricter monitoring for GDM from early pregnancy, while in obese women, recommended GWG may need to be re-evaluated, since refraining from any weight gain may have a preventive effect for GDM.

## 1. Introduction

Gestational Diabetes Mellitus (GDM) is a growing pandemic with numerous complications and an increasing healthcare cost [1,2]. GDM is a metabolic condition with a variable degree of carbohydrate intolerance, first diagnosed in pregnancy [3]. Although there is a wide heterogeneity for the criteria used for its diagnosis, the global prevalence of GDM is continuously rising due to the increase in obesity and the maternal childbearing age [4]. GDM imposes a medical alert for both the mother and the fetus as it is highly associated with adverse obstetric and perinatal outcomes [5]. Maternal outcomes include proximate complications, such as cesarean delivery, and future metabolic disorders, such as metabolic syndrome and type 2 diabetes mellitus [6,7,8,9]. Fetal complications that are related to GDM include fetal overgrowth leading to large for gestational age (LGA) neonates, respiratory distress syndrome (RDS), preterm birth, and stillbirth [10,11]; there are also looming future risks for obesity, cardiovascular disease, glucose intolerance, and learning difficulties [12,13,14].

Gestational weight gain (GWG) is an indicator of maternal fat accumulation, an essential component of fetal growth and of a healthy pregnancy course [15]. However, due to the fact that excess body fat and pre-pregnancy obesity have been closely related to GDM [16], the Institute of Medicine (IOM) proposed guidelines in 2009 on the optimal GWG during pregnancy with range estimates in order to minimize related adverse perinatal outcomes [17]. These guidelines are suggestions according to the pre-pregnancy BMI and it remains unclear whether they are applicable to high-risk pregnancies, such as those with clinical pre-pregnancy obesity and/or women diagnosed with GDM [18]. It is however known that excessive GWG, i.e., beyond compliance to the IOM guidelines, has been reported to be associated with GDM-related adverse perinatal outcomes, such as cardio-metabolic events, caesarean delivery, fetal overgrowth, and preterm birth [19]; nevertheless, specific high-risk subgroups by weight status have not been well investigated.

Results from a meta-analysis indicated that there is an association between increasing maternal BMI and the occurrence of GDM [20]. The authors, however, stated that results may be biased, because many studies did not adjust for confounders, such as maternal age (MA), which are strongly correlated with increasing BMI and risk of GDM. Moreover, a recent comparative review reported on the significant differences among the National and International Guidelines on the issue of screening for GDM [21]. Hence, more studies are needed to elucidate this relationship. Additionally, compared to women with normal glucose tolerance (NGT), those who are diagnosed with GDM already have a subclinical metabolic dysregulation before conception [22]. This predisposing metabolic malfunction is further aggravated and, in combination with beta cell malfunction, leads to the occurrence of GDM, as a result of the considerable decrease in insulin sensitivity that occurs in a typical pregnancy. Moreover, heterogeneity in the extent of hyperglycemia across GDM individuals may be present [23].

Many studies highlight the importance of closely monitoring high-risk populations by measuring the GWG, as it may be a useful tool to early diagnose, prevent or even control the occurrence and outcomes of GDM [24,25,26]. The aim of this study was to examine the association between GWG and GDM likelihood, overall, and by pre-pregnancy weight status.

## 2. Materials and Methods

### 2.1. Variables and Population Characteristics

All data were collected from eligible study participants attending the 3rd Department of Obstetrics and Gynaecology, Aristotle University of Thessaloniki, Greece. The study received approval by the Bioethics Committee of the Aristotle University of Thessaloniki, Greece (6.231/29.7.2020). Eligibility criteria were (i) age > 18 years old, (ii) absence of a serious medical condition (such as renal disease or hypertension), (iii) absence of pre-existing diabetes (type 1 or type 2), (iv) known weight and height at 2 pre-defined time points (before conception and at the 20^+0^–23^+6^-week visit). Maternal characteristics were collected, and included MA, height, and weight pre-pregnancy. Height was measured in cm with the use of a stadiometer and weight was retrieved from clinical records. Past medical history, parity, gravidity, and past and current smoking status were also recorded.

The criteria used for the diagnosis of GDM were from the Hellenic Society of Obstetricians and Gynaecologists (HSOG), based on the Hyperglycemia and Adverse Pregnancy Outcome (HAPO) study [27]. In our dataset, we recruited women who attended the antenatal clinic for their ultrasound screening at 11^+0^–13^+6^ weeks of gestation. Early diagnosis of GDM (<24 weeks of gestation) is usually performed with a blood glucose screening at 7–9 weeks of gestation, so those women were not included in our analysis. Briefly, women underwent a measurement of blood glucose concentration while fasting for at least 8 h, and then an Oral Glucose Tolerance Test (OGTT) of 75 g oral glucose was administered, at 24–28 weeks of gestation. Diagnosis for GDM was set when at least one of the following was present: fasting blood glucose ≥92 mg/dL, blood glucose concentration at one hour post OGTT ≥ 180 mg/dL, and/or blood glucose concentration 2 h post OGTT ≥ 153 mg/dL [28].

### 2.2. Calculation of Weight Gain

GWG was calculated as the difference between the weight at the 20^+0^–23^+6^ week visit (Wt now) and the baseline weight before pregnancy (Wt pre), retrieved from clinical records. The body mass index (BMI) was computed using the height (m) and weight (kg) of participants in the formula BMI = kg/m^2^. The pre-pregnancy (BMI pre) was calculated using the “Wt pre” and height, while “Wt now” and height were used to calculate BMI at the 20^+0^–23^+6^ week visit (BMI now). Participants were then further divided into subgroups as per their pre-pregnancy BMI status, based on the following BMI classification criteria: (UW) underweight BMI < 18.5 kg/m^2^; (NW) normal weight BMI 18.5–24.9 kg/m^2^; (OW) overweight BMI 25–29.9 kg/m^2^; and obese BMI ≥ 30 kg/m^2^; whereas obese individuals were further categorized into levels of obesity: obese, OB1 (BMI 30–34.9 kg/m^2^); clinical obesity: OB2 (BMI 35–39.9 kg/m^2^); and morbid obesity: OB3 (BMI ≥ 40 kg/m^2^) [29].

### 2.3. Estimation of Expected Weight Gain Ranges

Based on the exact gestational age of each participant at the 20^+0^–23^+6^ week visit, we calculated the minimum, normal, and maximum recommended weight gain based on the IOM guidelines. Then, based on their actual weight change, each participant was categorized in one of the following categories: “Wgcat less” when the weight gain from pre pregnancy to the 20^+0^–23^+6^ week visit was below the lower limit of the suggested weight gain range, “Wgcat normal” when the weight gain was within the range proposed by the IOM, and “Wgcat more” when the weight gain was more than the highest cut off.

Moreover, for individuals in the “Wgcat more” category, i.e., those who gained more weight than the upper limit suggested by IOM, we further calculated the excess weight gain (WGextra). Then, we divided the extra weight gain per kg and created categorical variables to test if the excessive weight gain per each kg played a role in the occurrence of GDM.

### 2.4. Statistical Analysis

For continuous variables, normality was assessed using the Shapiro–Wilk test, variance equality with the F-test, and the hypothesis testing was performed using either *t-*test, Wilcoxon, or Mann–Whitney test, depending on variable distribution results. For binary variables, the Fisher’s exact test was used, depending on the number of samples. Regarding the final model for the calculation of the adjusted odds ratio, a Generalized Linear Model was used, with a binomial likelihood (for the binary variable GDM), and a logit link function. All statistical implementations were performed in the R language (v4.2.1).

## 3. Results

The general characteristics of the population are presented in Table 1. Mean MA, Wt pre, BMI pre and mean GWG were higher in the GDM group compared to non-GDM cases. Results from crude analysis indicated that MA (W statistic: 1.99, 95%CI: [1.39, 2.60], *p* < 0.0001), BMI pre (W statistic: 2.20, 95%CI: [1.70, 2.70], *p* < 0.0001) and BMI now (W statistic: 2.36, 95%CI: [1.84, 2.89], *p* < 0.0001) were found to be higher in women with GDM and this may be explained as a probable causal relationship. On the other hand, GWG in the overall population was not found to be associated with the occurrence of GDM (W statistic: 7.01, 95%CI: [−5.73, 0.30], *p* = 0.95).

The likelihood of GDM was lower in the NW pre group (OR: 0.49 [0.39, 0.62], *p* < 0.0001), and higher in the OW pre (OR: 1.36, 95%CI: [1.03, 1.77], *p* = 0.021) and in all the obesity categories: OB1 pre (OR: 2.40, 95%CI: [1.72, 3.29] *p* < 0.0001), OB2 pre (OR: 2.08, 95%CI: [1.16, 3.51], *p* = 0.008), and OB3 pre (3.98 [1.84–7.86], *p* = 0.0003).

The incidence of GDM in the NW now population was lower (OR: 0.43, 95%CI: [0.33, 0.55], *p* < 0.0001), whereas it was higher in OB1 now (OR: 1.89, 95%CI: [1.41, 2.50], *p* < 0.0001), OB2 now (OR: 2.82, 95%CI: [1.14, 2.83], *p* = 0.008), or OB3 now cases (OR: 4.78, 95%CI: [2.73, 8.03], *p* < 0.0001). 

Additionally, crude analysis indicated that GDM occurrence was higher in women who underwent conception with assisted reproductive technology (ART) methods (OR: 2.08, 95%CI: [1.34, 3.13], *p* = 0.01) but when adjusted for confounders there was no significance (aOR: 1.15, 95%CI: [0.73, 1.77], *p* = 0.51). 

Women of age greater than 35 years old had higher rates of GDM incidence (OR: 1.75, 95%CI: [1.37, 2.22], *p* < 0.0001). Moreover, GWG extra >8 kg was found to be significant for GDM occurrence in general (OR: 2.04 [1.30, 3.11], *p* = 0.01). 

The GWG categories for the different subgroups of the entire population are present-ed in Table 2. Women in the non-GDM population were more likely to have normal weight BMI before pregnancy in comparison to the GDM group (*p* < 0.001). Women who developed GDM were more likely to belong in the category that gained weight above the expected ranges (OB1 15.1% vs. 7.2% *p* = 0.0002, and OB2 5.3% vs. 1.96% *p* = 0.004). The mean weight changes for the different subgroup BMI categories are presented in Table 3. Notably, only 24% of the entire population gained weight within the expected ranges according to IOM (Supplementary Material Figure S3). In the GDM group, NW and OB2 had greater weight change in comparison to the other subgroups. In the non-GDM group the UW population had more weight change, followed by the NW group. The lowest weight change was observed in the OB3 category, in both the GDM and non-GDM groups.

We also performed a subgroup crude analysis for the outcome GDM by BMI categories (UW, NW, OW, OB1, OB2, or OB3) (Table 4). Specifically, in the NW category, Wt pre, Wt now, and BMI now, were higher in the GDM group [(W statistic: 1.99, 95%CI: [0.99, 2.99], *p* = 0.03), (W statistic: 2.00, 95%CI: [0.99, 3.00], *p* = 0.02), (W statistic: 0.79, 95%CI: [0.42, 1.16], *p* = 0.0002) respectively]. In OB2 individuals with GDM, BMI now was higher in the GDM group (W statistic: 3.16, 95%CI: [0.69, 2.99], *p* = 0.03).

We computed the aOR using different GLMs, resulting in different adjustments (Table 5). Different subgroups of the population were examined related to whether the participants belonged to the Wgcat less, Wgcat normal or Wgcat more categories, adjusted for MA, gravidity, smoking, thyroid disease (hyperthyroidism, hypothyroidism, Hashimoto’s disease), BMI pre and conception method. The results demonstrate that women who developed GDM and belonged in the Wgcat less (women who gained less weight compared to the minimum expected), had higher MA and BMI pre when compared to the non-GDM individuals (aOR:1.05, 95%CI: [1.003, 1.11], aOR: 1.08, 95%CI: [1.04, 1.12] respectively). The same confounders were observed to play a role for women who developed GDM and gained weight within the normal expected ranges [MA (aOR: 1.08, 95%CI: [1.02, 1.15], BMI pre (aOR: 1.09, 95%CI: [1.04, 1.14]). In women who gained weight more than the upper expected ranges (Wgcat more), the incidence of GDM, MA, and BMI pre were higher, in comparison to the non-GDM groups.

Subgroup analysis was performed specifically for each BMI category before pregnancy, adjusted for MA, gravidity, smoking, thyroid disease, BMI pre and conception method (Table 6). The findings indicate that in all subgroups except OB2 pre, MA was higher in the GDM group, while GWextra in the OB2 pre group was higher in the GDM individuals (aOR1.12, 95%CI: [1.01, 1.26]). Moreover, women in the NW pre group that developed GDM had higher BMI pre (aOR: 1.24, 95%CI: [1.13, 1.37]).

The subgroups for each BMI category at mid-gestation, adjusted for MA, gravidity, smoking, thyroid disease, BMI pre and ART were computed. Increasing MA and BMI pre in the group that was within the normal BMI range (NW now) following the weight changes that occurred until mid-gestation showed that there was a significant association with the occurrence of GDM (aOR: 1.21, 95%CI: [1.06, 1.38], aOR: 1.09, 95%CI: [1.05, 1.14], respectively). In the OW now group, MA and BMI pre were associated with a higher incidence of GDM (aOR: 1.06, 95%CI: [1.02, 1.11], aOR: 1.12, 95%CI: [1.02, 1.23], respectively) (Table 7).

Women who were at OB1 category at mid- gestation (OB1 now) and were of advanced age (>35 years), had higher rates of GDM development (aOR: 1.10, 95%CI: [1.04, 1.16]). Women in the OB3 category at mid-gestation who were diagnosed with GDM had overall gained less weight than expected (aOR: 5.00, 95%CI: [1.14, 23.69]), compared to the IOM, and had a higher MA (aOR: 1.19, 95%CI: [1.04, 1.39]). A limited number of women were classified as underweight at mid-gestation (UW now); hence no further analysis could be performed to draw specific conclusions. In most of the adjusted odds ratio calculations (Table 7), MA plays an important role in the appearance of GDM, in all the subgroups except for the OB2. For the OB2 now category, no characteristic was identified of playing an important role for GDM occurrence. As the women increased their weight category, the BMI pre played less role in the GDM occurrence, i.e., it played no role from OB and above where only MA is important, except for the OB2 subgroup.

The BMI classification shifts from pre pregnancy to mid-gestation are shown in Table 8. Based on the pre-pregnancy BMI category, women in the NW pre group who remained NW, according to the IOM, at mid-gestation (NW now), had less risk of GDM occurrence (OR: 0.60, 95%CI: [0.43, 0.85]). On the contrary, the population within the NW pre group that gained weight above the recommendations and shifted their BMI category to the “OW now” or “OB1 now” categories, had a higher incidence of GDM (OR: 1.52, 95%CI: [1.08, 2.14], OR: 4.38, 95%CI: [1.08, 13.13]). On the other hand, for the individuals in the OB1 pre group who remained within the same category (OB1 now) or shifted their category to OB2, no significant association for the GDM outcome was observed (OR: 0.77, 95%CI: [0.41, 1.46], OR: 1.19, 95%CI: [0.61, 2.26], respectively). Moreover, OB2 individuals who remained OB2 had a lower rate of GDM occurrence (OR: 0.26, 95%CI: [0.07, 0.81]), while a shift from the OB2 to OB3 BMI category was associated with higher incidence of GDM (OR: 4.85, 95%CI: [1.50, 15.95]). Furthermore, based on the calculations for the extra weight gain for the different BMI subgroups, in the NW group, we found a higher association of GDM occurrence when the extra weight gain exceeded 8 kgs (OR: 2.13, 95%CI: [0.98, 4.21], *p* = 0.03), while for the OB2 population, the excess weight gain seemed to be significant when it was more than 11 kgs (OR: 29.39, 95%CI: [2.20, 1611.15], *p* = 0.004).

Additionally, we incorporated figures to depict the BMI pre status for the GDM and non-GDM groups, as well as, for the WG extra, BMI pre in relation to which weight gain category the entire group (GDM and non-GDM) belonged (Wgcat less, Wgcat normal, Wgcat more). This material is in the section “Supplementary Material”. In all figures, BMI pre is lower in the non-GDM group (Supplementary Materials Figures S1–S5).

## 4. Discussion

This study showed that 1. increased MA and high BMI before pregnancy increases the odds for GDM; 2. GWG was higher than the recommended by IOM in the GDM group compared to the non-GDM group; 3. women who were NW pre and remained NW by mid-gestation had lower rates of GDM, while those who moved to a higher BMI category (overweight or obese) were at higher risk for developing GDM; 4. women who conceived while being OB2 (OB2 pre) had a lower risk of developing GDM if they remained within the same BMI category by mid-gestation, while shifting to OB3 increased their risk of GDM; the risk was higher when the weight gain limit was exceeded by more than 11 kg; 5. only one in four women of the entire population gained weight within the expected ranges according to IOM.

Our findings on the association between MA and pre pregnancy BMI with GDM are in accordance with those reported in the literature. A recent meta-analysis by Li et al. which included 120 million participants demonstrated that MA increases the risk of GDM linearly. More specifically, in Caucasians, the authors estimated that for each year of increase in MA, since the age of 18 years, the risk for GDM increases by 12.7%; the risk increased even more if the women were Asian and older than 25 years old [30]. Another recent meta-analysis indicated that maternal obesity was associated with an increased risk of GDM, especially in visceral obesity [31]. Although several studies indicate a higher risk for developing GDM with increasing maternal weight/BMI, the extent to which these conditions converge remains ambiguous as there are many confounders that should be considered. Moreover, the non-unanimous use of diagnostic criteria for GDM and the different variations of the reported GDM prevalence may obscure the greater image [32]. 

The association between GWG and GDM, found in our study, is also in agreement with previous investigators. A study by Gibson et al. also reported the significance of weight gain as a contributor to the occurrence of GDM, in individuals who were overweight or obese before pregnancy [33]. Moreover, in our study, individuals who had normal pre pregnancy BMI (NW pre) but shifted their BMI category to either OW or OB1 had higher risk for developing GDM. Excess weight gain during pregnancy has been numerously associated with GDM occurrence and risk for unfavorable delivery outcomes [34,35,36,37]. Additionally, in our study, OB2 individuals who did not have excessive GWG had lower rates of GDM occurrence, while switching from OB2 to OB3 by mid-gestation was associated with higher incidence of GDM. As indicated by our results, BMI pre status in the OB2 population seemed to be an important risk factor for GDM development. A recent network meta-analysis on preventive methods for GDM development, including GWG in an obese population indicated that none of the preventive methods used (diet and/or physical activity, or medication), managed to reduce the risk for GDM, although physical activity alone, diet alone, and diet and physical activity, were effective in preventing excess weight gain [38]. On the other hand, weight gain in the lower cut offs for the expected weight gain in the OB3 category was not protective for the development of GDM; this may be attributed to the excessive pre-pregnancy BMI, as this has been reported in literature as an independent risk factor for GDM occurrence, with higher significance compared to excessive weight gain [39].

Some studies report on the association between GWG adherence to the IOM guidelines in GDM women and adverse perinatal outcomes. More specifically, a study by Cheng et al. that analyzed data of 31,000 women with GDM reported that individuals who had GWG above the IOM guidelines had higher a incidence of preterm birth, cesarean delivery, and neonatal macrosomia [36]. On the other hand, women who had GWG below the IOM guidelines had higher incidents of small for gestational age (SGA) neonates. Moreover, when the total weight gain was below the IOM guidelines, women had a better glycemic control without increased risk for LGA. Additionally, GWG seemed to play an important role in glycemic control, especially in obese populations with GDM [40]. Regarding weight changes, a previous meta-analysis showed that women with normal pre-pregnancy BMI and GWG according to the IOM guidelines, had less complicated pregnancies compared to women who did not adhere [41]. Moreover, a study conducted by Benhalima et al. that assessed GDM subtypes, namely the degree of insulin resistance in GDM individuals according to the Matsuda index, and their influence on maternal characteristics such as BMI and their pregnancy outcomes, concluded that insulin-resistant GDMs had more rates of complicated pregnancies [42]. Specifically, the study reported that insulin-resistant GDM women had significantly higher BMI and fasting plasma glucose and had gained more weight in early pregnancy compared to GDMs with insulin-sensitive GDM.

Additionally, a few studies in the literature reported an association between the use of ART and the incidence of GDM. Many individuals with advanced MA and polycystic ovaries syndrome (PCOS) undergo ART methods to conceive. The relationship between PCOS and GDM is well established but inclusion of such individuals in studies for identification of ART-related GDM occurrence may be biased as PCOS predisposes to insulin resistance [43,44]. Nonetheless, results from a meta-analysis showed that the use of ART was found to be substantially higher among women with GDM, even when women with PCOS were excluded from the analysis. This could be attributed to the associated pathologies that affect fertility such as obesity and advanced MA. However, these analyses also included studies that did not adjust for confounding factors, and therefore the results may be titled towards the association of ART methods and GDM occurrence [45]. In our study, use of ART methods compared to spontaneous conception, in crude analysis, was associated with a higher rate of GDM. However, the aOR for ART case was found to be non-significant, so we cannot assume any association. This correlation needs to be further evaluated in other studies and meta-analyses.

Ultimately, although the IOM has provided a specific spectrum of guidelines for GWG, optimal range variations still remain under the scope of investigation. It should be noted that these recommendations may need further evaluation for specific high-risk pregnancy subgroups, such as the women with GDM. Studies that sought the association of GWG within or beyond the IOM recommendations in relation to the incidence of GDM had inconsistent findings. A study concluded that for GWG above the IOM guidelines, there was no association in UW, NW, or OB populations with GDM but found a lower incidence when women were OW [46]. Another study found increased risk of GDM in NW and decreased risk in OW [47]. Sin et al. reported that weight gain above the IOM guidelines was associated with lower risk for GDM in all subgroups except for the UW population [48]. Hence, more evidence is needed under the scope of investigation to optimize gestational weight change recommendations.

Finally, the identification of BMI shifts within the different BMI subgroups showed that GWG within the expected gestational age ranges was associated with fewer cases of GDM, while excess weight gain that caused a BMI shift towards its higher classifications was associated with higher incidence of GDM.

Non-invasive predictive models for GDM have been reported in the literature. Kumar et al. proposed a two-tier predictive model to assess risk factors for early prediction of GDM in an Asian population that is more efficient than the existing UK NICE guideline model [49]. Prediction models are a useful tool that allows testing for numerous variables and confounders and could possibly be widely implemented in healthcare facilities to provide effective screening not only for the high-risk population but also for the general population.

Excessive weight prior to conception predisposes an individual to the occurrence of GDM, as has been previously reported [35]. Overweight or obesity before pregnancy or excessive weight gain during pregnancy, independently of GDM, is related to serious maternal and fetal complications [50]. The range of thresholds and the role of weight gain or loss among different subgroups need to be further elucidated. Overweight and obese women of reproductive age should be motivated for interventions aimed at reducing weight before pregnancy, either by following a healthy nutrition plan or by incorporating physical activity. Additionally, overweight or obese women with the diagnosis of GDM should follow an assisted plan for effective weight management during pregnancy. Moreover, women who remain overweight or obese during postpartum should also follow an attending plan for weight loss. As Glazer et al. pointed out, women who retained extra weight after delivery but achieved weight loss between pregnancies had lower rates of developing GDM [51]. Thus, effective lifestyle interventions, for women of reproductive age should be a priority to restrain adverse complications.

To our knowledge, this is the first study conducted in Northern Greece that investigated the association of weight changes during pregnancy on the occurrence of GDM, and it may be used as a future reference for the Greek population. Unlike other studies, this study included all BMI subgroups and performed additional subgroup analysis to compare weight changes to the expected weight gain ranges for gestational age. The number of studies examining all subgroups within a specific population, and those stratifying their results based on the different obesity degrees is limited, while in many other studies, usually a specific subgroup category would be analyzed. Additionally, this study used the data to create a prediction model for GDM screening. Results were additionally adjusted for confounders such as maternal age and BMI that are strong predisposing factors for GDM occurrence. Furthermore, an addition to this study is that it analyzed the effect of each separate excessive weight gain for each subgroup category, adjusting for confounders on the outcome of GDM. Moreover, all participants were monitored at a single center, GDM diagnosis was performed according to specific criteria, and data were collected uniformly. However, the study had some limitations. Subgroups did not all have many participants and GDM cases. Due to the small number of GDM events, the GLM may suffer with bias, and as such, the results may be tilted towards the non-GDM group.

Additionally, the GLM is a limited model, in the sense that it only measures linear relationships. In future work, we will try to collect more GDM individuals to verify our results.

In summary, this study has pointed out the need to implement methods to lower the risk of developing GDM. Since there are many risk factors that may be amendable to intervention, more data on different races and ethnic groups are essential.

## 5. Conclusions

For women of reproductive age that begin their pregnancy in an advanced stage of age, closer monitoring from the early stages of pregnancy is recommended. Additionally, in the obese population the suggested GWG cut offs may require further evaluation as refraining from any weight gain may have a preventative impact on GDM. 

## Figures and Tables

**Table 1:** Characteristics of the included population (*n* = 5948).

	GDM (*n* = 325)	Non-GDM (*n* = 5623)	Statistic [CI] for Incidence or Likelihood of GDM	*p*-Values
MA (years) mean ± sd	33.7 ± 5.47	31.6 ± 5.25	1.99 [1.39, 2.60]	**<0.0001**
MA > 35 years old (*n*%)	124 (38.2%)	1464 (26%)	1.75 [1.37, 2.22]	**<0.0001**
Smoking (*n*%)	48 (14.8%)	759 (13.5%)	1.18 [0.79, 1.52]	0.50
Thyroid disease (*n*%)	19 (5.85%)	348 (6.19%)	1.19 [0.55, 1.51]	0.90
Parity (*n*%)	142 (43.7%)	2665 (47.4%)	0.86 [0.68, 1.08]	0.20
Gravidity mean ± sd	0.55 ± 0.7	0.61 ± 0.78	−5.34 × 10^−5^ [−2.18, 8.15]	0.41
Conception with ART (*n*%)	29 (8.92%)	252 (4.48%)	2.08 [1.34, 3.13]	**0.01**
Wt pre (kg) mean ± sd	72.6 ± 16.6	65.6 ± 13.6	5.99 [4.50, 7.00]	<**0.0001**
BMI pre mean ± sd	26.5 ± 5.75	23.9 ± 4.7	2.20 [1.70, 2.70]	<**0.0001**
UW pre (*n*%)	10 (3.08%)	292 (5.19%)	0.57 [0.27, 1.09]	0.11
NW pre (*n*%)	153 (47.1%)	3615 (64.3%)	0.49 [0.39, 0.62]	<**0.0001**
OW pre (*n*%)	81 (24.9%)	1100 (19.6%)	1.36 [1.03, 1.77]	**0.021**
OB1 pre (*n*%)	53 (16.3%)	422 (7.5%)	2.40 [1.72, 3.29]	<**0.0001**
OB2 pre (*n*%)	17 (5.23%)	145 (2.58%)	2.08 [1.16, 3.51]	**0.008**
OB3 pre (*n*%)	11 (3.38%)	49 (0.87%)	3.98 [1.84–7.86]	**0.0003**
GWG (kg) mean ± sd	6.57 ± 4.41	6.49 ± 3.93	7.01 [−5.73, 0.30]	0.95
UW now (*n*%)	2 (0.62%)	32 (0.57%)	1.08 [0.12, 4.27]	0.79
NW now (*n*%)	90 (27.7%)	2647 (47.1%)	0.43 [0.33, 0.55]	<**0.0001**
OW now (*n*%)	118 (36.3%)	1911 (34%)	1.10 [0.86, 1.40]	0.39
OB1 now (*n*%)	70 (21.5%)	713 (12.7%)	1.89 [1.41, 2.50]	<**0.0001**
OB2 now (*n*%)	25 (7.7%)	244 (4.34%)	2.82 [1.14, 2.83]	**0.008**
OB3 now (*n*%)	20 (6.15%)	76 (1.35%)	4.78 [2.73, 8.03]	<**0.0001**
BMI now mean ± sd	28.9 ± 5.8	26.4 ± 4.7	2.36 [1.84, 2.89]	<**0.0001**

Suffix “pre” refers to a measurement variable before conception in kg; Suffix “now” refers to a measurement variable at 20^+0^–23^+6^ weeks of gestation, until the screening for diagnosis for GDM; MA: maternal age; MA > 35: maternal age >35 years old; conception ART: conception with the methods of assisted reproductive technologies; Wt pre: weight pre-pregnancy in kg; BMI pre: body mass index pre-pregnancy in kg/m^2^; body mass index (BMI) classifications: (UW) underweight BMI < 18.5 kg/m^2^, (NW) normal weight BMI 18.5–24.9 kg/m^2^, (OW) overweight BMI 25–29.9 kg/m^2^ and obese BMI > 30 kg/m^2^. Obese individuals were further categorized into OB1 (BMI 30–34.9 kg/m^2^), OB2 (BMI 35–39.9 kg/m^2^), OB3 (BMI ≥ 40 kg/m^2^); GWG: weight gain during 1st half of pregnancy (until 20^+0^–23^+6^ gestational weeks, before diagnosis of GDM); For continuous variables, a two-sample *t*-test or Mann–Whitney was used, depending on if the variable was normally distributed or not (Shapiro–Wilk test); all (*n*%) are odds ratios, whereas all values presented as mean ± sd are W statistic; for binary variables a two-sample Fisher’s exact test was utilized.

**Table 2:** Gestational weight gain category for the different BMI subgroups classification for the overall population (GDM, non-GDM).

	Gestational Weight Gain Category								
	Wgcat Less *n* (%)			Wgcat Normal *n* (%)			Wgcat More *n* (%)		
	GDM	Non-GDM	*p* Value	GDM	Non-GDM	*p* Value	GDM	Non-GDM	*p* Value
UW pre	3 (5%)	46 (5.5%)	1	3 (5.08%)	92 (6.72%)	0.79	4 (1.94%)	154 (4.51%)	0.11
NW pre	21 (35%)	483 (57.7%)	**0.0007**	29 (49.2%)	967 (70.6%)	**0.0008**	103 (50%)	2165 (63.4%)	**0.0001**
OW pre	13 (21.7%)	162 (19.4%)	0.61	14 (23.7%)	177 (12.9%)	**0.03**	54 (26.2%)	761 (22.3%)	0.2
OB1 pre	12 (20%)	80 (9.56%)	**0.02**	10 (17%)	95 (6.94%)	**0.009**	31 (15.1%)	247 (7.23%)	**0.0002**
OB2 pre	4 (6.67%)	48 (5.73%)	0.77	2 (3.39%)	30 (2.19%)	0.38	11 (5.34%)	67 (1.96%)	**0.004**
OB3 pre	7 (11.7%)	18 (2.15%)	**0.0008**	1 (1.69%)	8 (0.58%)	0.31	3 (1.46%)	23 (0.67%)	0.18
Total	60	837	-	59	1369	-	206	3417	-

BMI classification: (UW) underweight BMI <18.5 kg/m^2^, (NW) normal weight BMI 18.5–24.9 kg/m^2^, (OW) overweight BMI 25–29.9 kg/m^2^ and obese BMI > 30 kg/m^2^. Obese individuals were further categorized into (OB1) BMI 30 to <35 kg/m^2^, (OB2) BMI 35 to <40 kg/m^2^, (OB3) BMI ≥ 40 kg/m^2^; Wgcat less: weight gain below the cut offs for gestational age; Wgcat normal: weight gain within the cut offs for gestational age; Wgcat more: weight gain above the cut offs for gestational age.

**Table 3:** Mean weight gain in all subgroups of the two groups.

	Mean Weight Changes for All Subgroups (kg)				
	GDM (Ν = 325)		Non-GDM (Ν = 5623)		
UW	**10**	6.4 ± 3.34	292	7.26 ± 3.68	
NW	153	7.22 ± 3.94	3616	6.79 ± 3.43	
OW	81	6.84 ± 4.43	1100	6.36 ± 4.19	
OB1	53	5.16 ± 4.64	422	5.34 ± 5.01	
OB2	17	7.06 ± 6.22	145	3.48 ± 5.26	
OB3	11	1.73 ± 3.2	49	1.71 ± 7.69	

BMI classification: UW (underweight, BMI < 18.5 kg/m^2^), NW (normal weight, BMI 18.5–24.9, OW (overweight, BMI 25.0–29.9 kg/m^2^).

**Table 4:** Subgroup crude analysis for underweight, normal weight, and overweight individuals based on BMI classification for outcome GDM.

	UW		NW		OW		OB1		OB2		OB3		
	Statistic [CI]	*p*	Statistic [CI]	*p*	Statistic [CI]	*p*	Statistic [CI]	*p*	Statistic [CI]	*p*	Statistic [CI]	*p*	Statistic Name
MA > 35 years	3.54 [0.7, 15.6]	1	1.72 [1.21, 2.44]	0.13	1.51 [0.90, 2.48]	1	1.30 [0.67, 2.48]	1	1.90 [0.59, 5.96]	1	4.25 [0.91, 23.1]	1	Odds ratio
Parity	0.74 [0.12, 3.33]	1	0.77 [0.54, 1.09]	0.53	0.68 [0.42, 1.10]	1	0.73 [0.39, 1.36]	1	1.09 [0.35, 3.59]	1	1.97 [0.40, 13.0]	1	Odds ratio
Hypothyroidism	-	-	1.46 [0.56, 3.18]	0.92	1.69 [0.13, 2.20]	1	0.43 [0.01, 2.85]	1	1.00 [0.10, 4.95]	1	1.93 [0.16, 14.4]	1	Odds ratio
Wt pre	0.05 [−2.59, 2.73]	0.97	**1.99** **[0.99, 2.99]**	**0.003**	1.76 [−1.0, 1.99]	0.86	1.00 [−0.99, 3.99]	0.84	−0.99 [−5.00, 3.90]	0.66	−0.20 [−8.99, 7.33]	0.95	W statistic
Wt now	−0.41 [−4.52, 2.95]	0.97	**2.00** **0.99, 3.00]**	**0.002**	0.99 [−1.0, 2.99]	0.77	1.00 [−1.0, 4.0]	0.84	0.99 [−2.64, 8.05]	0.38	3.77 [−7.00, 6.99]	0.95	W statistic
BMI now	−0.39 [−1.46, 0.73]	0.97	**0.79** **[0.42, 1.16]**	**0.0002**	0.33 [−0.13, 0.75]	0.44	0.21 [−0.45, 0.85]	0.84	**3.16** **[0.69, 2.99]**	**0.03**	−0.53 [−2.08, 1.20]	0.95	W statistic
GWG	−0.99 [−3.00, 1.50]	0.97	1.07 [−6.65, 0.99]	0.20	3.00 [−0.99, 1.00]	0.77	−2.60 [−1.99, 1.00]	0.84	2.60 [0.87, 6.30]	0.06	−1.00 [−4.99, 1]	0.95	W statistic

**Table 5:** Subgroup analyses for the different weight gain categories (less, normal, more) adjusted to MA, gravidity, smoking, thyroid disease, BMI pre, and ART.

	Weight Gain Categories		
	Wgcat Less	Wgcat Normal	Wgcat More
WGextra	1.09 [0.98, 1.24]	0.87 [0.59, 1.27]	1.03 [0.98, 1.07]
MA	**1.05 [1.003, 1.11]**	**1.08 [1.02, 1.15]**	**1.09 [1.05, 1.12]**
Gravidity	0.87 [0.59, 1.24]	0.96 [0.67, 1.31]	**0.65 [0.51, 0.80]**
Smoking	1.004 [0.37, 2.28]	1.44 [0.67, 2.83]	0.98 [0.65, 1.45]
Thyroid disease	0.32 [0.05, 1.12]	0.57 [0.13, 1.61]	0.99 [0.53, 1.69]
BMI pre	**1.08 [1.04, 1.12]**	**1.09 [1.04, 1.14]**	**1.09 [1.06, 1.12]**
Conception ART	0.83 [0.18, 2.57]	1.54 [0.47, 4.10]	1.10 [0.62, 1.84]

WGextra: weight gain above the higher cut offs for gestational age; MA: maternal age; thyroid disease: includes hyperthyroidism, hypothyroidism and Hashimoto’s disease; BMI pre: body mass index pre-pregnancy; conception ART: conception with assisted reproductive technology methods; Wgcat less: weight gain below the cut offs for gestational age; Wgcat normal: weight gain within the cut offs for gestational age; Wgcat more: weight gain above the cut offs for gestational age; multivariate logistic regression with binomial likelihood was used to compute the adjusted odds ratios.

**Table 6:** Subgroup analyses for each BMI category before pregnancy, adjusted for MA, gravidity, smoking, thyroid disease, BMI pre, and ART.

	UW Pre	NW Pre	OW Pre	OB1 Pre	OB2 Pre	OB3 Pre	Total Population
Wgcat less	3.37 [0.65, 14.71]	1.09 [0.66, 1.73]	1.18 [0.60, 2.14]	1.27 [0.61, 2.49]	0.63 [0.15, 2.10]	4.17 [0.82, 25.56]	1.18 [0.86, 1.58]
Wgcat normal	1.07 [0.22, 4.13]	0.67 [0.43, 1.00]	1.09 [0.57, 1.94]	0.86 [0.39, 1.73]	0.55 [0.08, 2.30]	0.34 [0.01, 2.67]	0.78 [0.57, 1.03]
Wgcat more	0.43 [0.10, 1.63]	1.28 [0.91, 1.83]	0.85 [0.52, 1.41]	0.93 [0.51, 1.69]	1.98 [0.66, 6.48]	0.40 [0.05, 2.24]	1.07 [0.85, 1.36]
WGextra	0.85 [0.68, 1.03]	1.01 [0.97, 1.06]	1.01 [0.95, 1.06]	0.99 [0.93, 1.04]	**1.12 [1.01, 1.26]**	0.97 [0.86, 1.12]	1.01 [0.99, 1.04]
MA	**1.18 [1.02, 1.39]**	**1.09 [1.05, 1.12]**	**1.07 [1.02, 1.13]**	**1.05 [1.00, 1.12]**	1.06 [0.96, 1.19]	**1.26 [1.04, 1.63]**	**1.08 [1.06, 1.11]**
Gravidity	0.94 [0.28, 2.47]	**0.67 [0.51, 0.86]**	0.74 [0.53, 1.01]	0.77 [0.51, 1.10]	0.89 [0.43, 1.60]	0.54 [0.16, 1.60]	**0.75 [0.63, 0.88]**
Smoking	1.83 [0.34, 8.27]	0.80 [0.44, 1.34]	1.00 [0.49, 1.89]	1.14 [0.53, 2.28]	1.94 [0.48, 6.67]	1.91 [0.39, 9.70]	1.06 [0.76, 1.45]
Thyroid disease	3.68 [0.18, 26.94]	0.84 [0.39, 1.61]	0.39 [0.09, 1.10]	0.60 [0.09, 2.11]	0.89 [0.13, 3.60]	2.06 [0.24, 13.11]	0.73 [0.43, 1.16]
BMI pre	0.86 [0.38, 2.36]	**1.24 [1.13, 1.37]**	1.09 [0.92, 1.29]	1.04 [0.84, 1.28]	1.36 [0.93, 2.00]	0.92 [0.63, 1.21]	**1.09 [1.07, 1.11]**
Conception ART	4.68 [0.21, 41.06]	1.11 [0.58, 1.99]	1.67 [0.67, 3.76]	0.54 [0.08, 2.12]	0.75 [0.03, 5.28]	0.29 [0.008, 4.29]	1.15 [0.73, 1.77]

WGextra: weight gain above the higher cut offs for gestational age; MA: maternal age; thyroid disease: includes hyperthyroidism, hypothyroidism and Hashimoto’s disease; BMI pre: body mass index pre- pregnancy; conception ART: conception with assisted reproductive technology methods; Wgcat less: weight gain below cut offs for gestational age; Wgcat normal: weight gain within the cut offs for gestational age; Wgcat more: weight gain above the cut offs for gestational age; body mass index classifications (BMI) classifications: (UW) underweight BMI < 18.5 kg/m^2^, (NW) normal weight BMI 18.5–24.9 kg/m^2^, (OW) overweight BMI 25–29.9 kg/m^2^ and obese BMI > 30 kg/m^2^. Obese individuals were further categorized into (OB1) BMI 30 to <35 kg/m^2^, (OB2) BMI 35 to <40 kg/m^2^, (OB3) BMI ≥40 kg/m^2^; Suffix pre refers to a measurement variable before conception in kg; Suffix now refers to a measurement variable at 20^+0^–23^+6^ weeks of gestation, until the screening for diagnosis for GDM; multivariate logistic regression with binomial likelihood was used to compute the adjusted odds ratios.

**Table 7:** Subgroup analyses for each BMI category at 20^+0^–23^+6^ weeks of gestation, adjusted for MA, gravidity, smoking, thyroid disease, BMI pre, and conception method ART.

	UW Now	NW Now	OW Now	OB1 Now	OB2 Now	OB3 Now
Wgcat less	-	1.17 [0.66, 1.98]	1.34 [0.70, 2.43]	1.35 [0.58, 2.98]	1.03 [0.20, 4.16]	**5.00 [1.14, 23.69]**
Wgcat normal	-	0.90 [0.55, 1.41]	0.65 [0.36, 1.10]	0.96 [0.45, 1.91]	1.24 [0.33, 3.79]	0.34 [0.01, 2.17]
Wgcat more	-	0.99 [0.63, 1.55]	1.19 [0.75, 1.93]	0.84 [0.43, 1.65]	0.80 [0.25, 2.65]	0.47 [0.12, 1.75]
WGextra	-	0.98 [0.90, 1.05]	0.99 [0.92, 1.05]	1.05 [0.97, 1.14]	0.97 [0.84, 1.10]	0.95 [0.84, 1.06]
MA	-	**1.09 [1.05, 1.14]**	**1.06 [1.02, 1.11]**	**1.10 [1.04, 1.16]**	0.99 [0.92, 1.07]	**1.19 [1.04, 1.39]**
Gravidity	-	**0.59 [0.40, 0.85]**	0.82 [0.63, 1.05]	**0.60 [0.41, 0.86]**	0.93 [0.52, 1.51]	0.80 [0.35, 1.76]
Smoking	-	1.16 [0.55, 2.20]	0.70 [0.36, 1.25]	1.28 [0.64, 2.40]	1.16 [0.40, 3.01]	1.56 [0.45, 5.13]
Thyroid disease	-	1.20 [0.45, 2.63]	0.47 [0.16, 1.08]	0.81 [0.23, 2.13]	0.38 [0.02, 1.98]	2.27 [0.41, 10.49]
BMI pre	-	**1.21 [1.06, 1.38]**	**1.12 [1.02, 1.23]**	1.05 [0.95, 1.16]	0.88 [0.74, 1.05]	0.96 [0.81, 1.11]
Conception ART	-	1.20 [0.51, 2.51]	0.89 [0.37, 1.89]	2.03 [0.80, 4.80]	0.64 [0.03, 3.9]	1.23 [0.11, 10.4]

WGextra: weight gain above the higher cut offs for gestational age; MA: maternal age; thyroid disease: includes hyperthyroidism, hypothyroidism and Hashimoto’s disease; BMI pre: body mass index pre-pregnancy; conception ART: conception with assisted reproductive technology methods; Wgcat less: weight gain below cut offs for gestational age; Wgcat normal: weight gain within the cut offs for gestational age; Wgcat more: weight gain above the cut offs for gestational age; body mass index classifications (BMI) classifications: (UW) underweight BMI < 18.5 kg/m^2^, (NW) normal weight BMI 18.5–24.9 kg/m^2^, (OW) overweight BMI 25–29.9 kg/m^2^ and obese BMI > 30 kg/m^2^. Obese individuals were further categorized into (OB1) BMI 30 to <35 kg/m^2^, (OB2) BMI 35 to <40 kg/m^2^, (OB3) BMI ≥ 40 kg/m^2^; Suffix pre refers to a measurement variable before conception in kg; Suffix now refers to a measurement variable at 20^+0^–23^+6^ weeks of gestation, until the screening for diagnosis for GDM; multivariate logistic regression with binomial likelihood was used to compute the adjusted odds ratios.

**Table 8:** Crude analysis of BMI changes at the time of screening for GDM.

BMI Shifts	BMI Classification Shifts at 20^+0^–23^+6^ Weeks of Gestation	
NW pre → NW now	0.60 [0.43, 0.85]	Lower in GDM
NW pre → OW now	1.52 [1.08, 2.14]	Higher in GDM
NW pre → OB1 now	4.38 [1.08, 13.13]	Higher in GDM
OB1 pre → OB1 now	0.77 [0.41, 1.46]	NS
OB1 pre → OB2 now	1.19 [0.61, 2.26]	NS
OB2 pre → OB2 now	0.26 [0.07, 0.81]	Lower in GDM
OB2 pre → OB3 now	4.85 [1.50, 15.95]	Higher in GDM

Body mass index classifications (BMI) classifications: (UW) underweight BMI <18.5 kg/m^2^, (NW) normal weight BMI 18.5–24.9 kg/m^2^, (OW) overweight BMI 25–29.9 kg/m^2^ and obese BMI > 30 kg/m^2^. Obese individuals were further categorized into (OB1) BMI 30 to <35 kg/m^2^, (OB2) BMI 35 to <40 kg/m^2^, (OB3) BMI ≥ 40 kg/m^2^; NS: not significant; two-sample *t*-test or Mann–Whitney was used, depending on if the variable was normally distributed or not (Shapiro–Wilk test).

## Data Availability

The data presented in this study are not publicly available due to privacy restrictions.

## Supplementary Materials

The following supporting information can be downloaded at: https://www.mdpi.com/article/10.3390/nu15040893/s1, Figure S1: BMI pre in GDM vs Control group; Figure S2: BMI pre in GDM and Control group for those who gained less weight; Figure S3: BMI pre in GDM and Control group for those who gained normal weight; Figure S4: BMI pre in GDM and Control group for those who gained more weight; Figure S5: BMI pre in GDM and Control group for those who gained extra weight.
