# Supplementary material for: Effect of Gestational Weight Gain during the First Half of Pregnancy on the Incidence of GDM, Results from a Pregnant Cohort in Northern Greece
Source: Nutrients. 2023 Feb 10;15(4):893. doi: 10.3390/nu15040893 (PMC9964795; doi:10.3390/nu15040893)

Suplementary Material

Suppl. Figure S1: BMI pre in GDM vs Control group

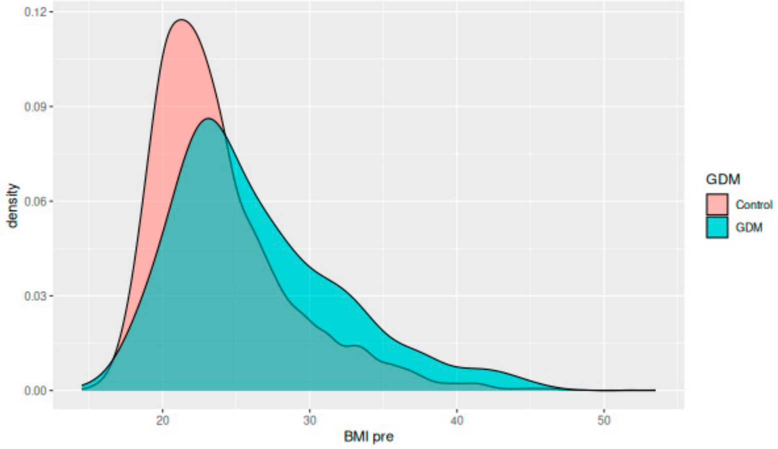

Suppl. Figure S2: BMI pre in GDM and Control group for those who gained less weight

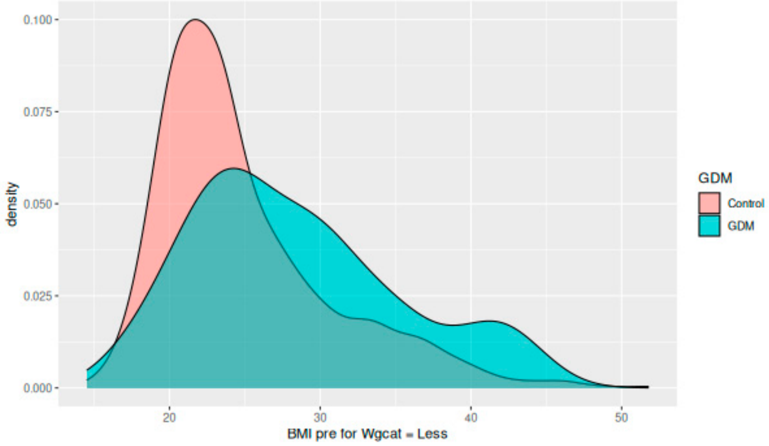

Sup. Figure S3: BMI pre in GDM and Control group for those who gained normal weight

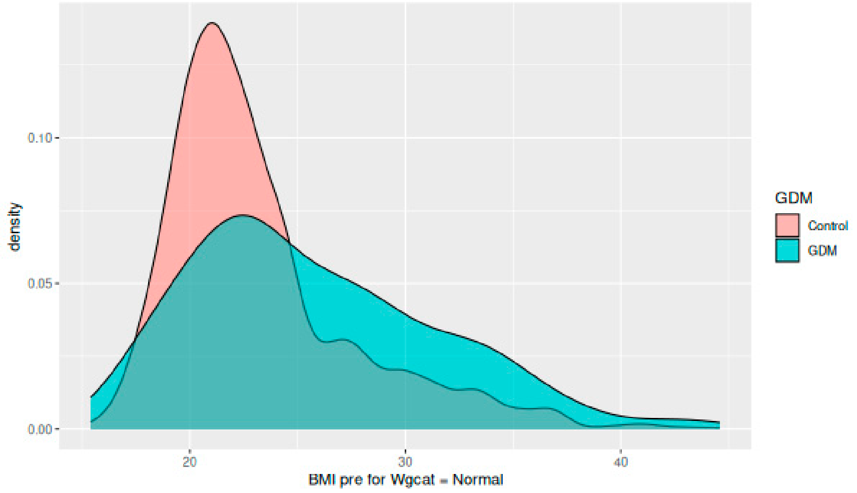

Supp. Figure S4: BMI pre in GDM and Control group for those who gained more weight

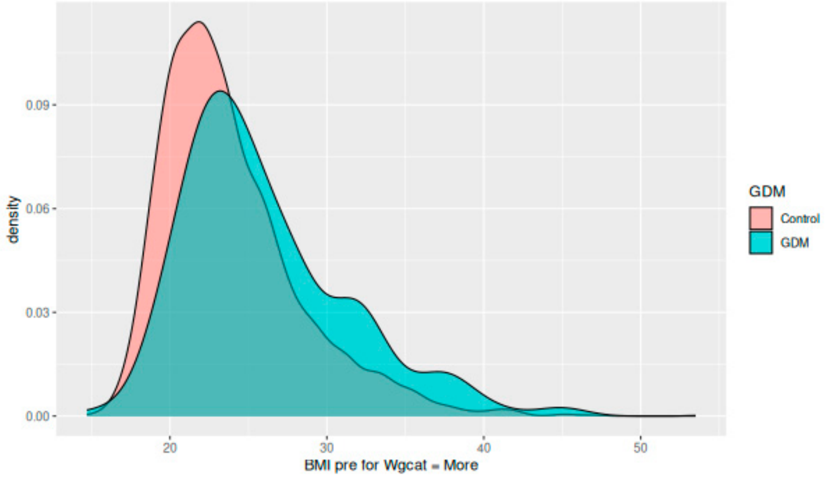

Suppl. Figure S5: BMI pre in GDM and Control group for those who gained extra weight

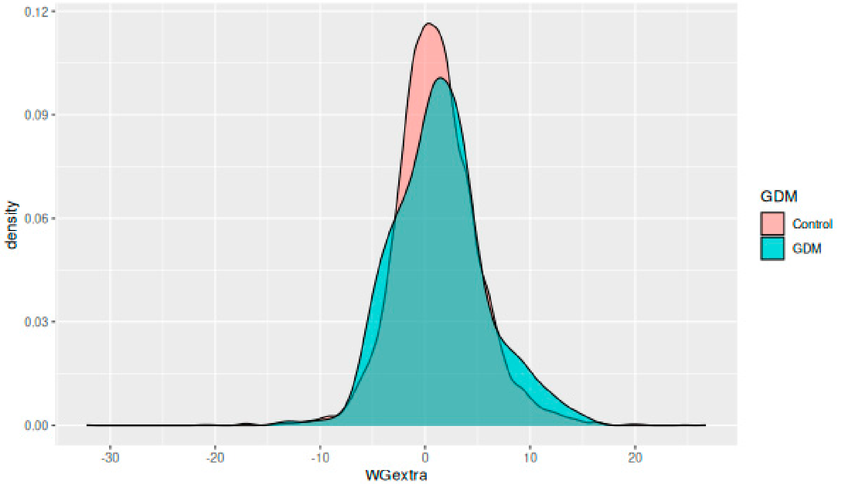

Supplement: Supplementary file 1 [file nutrients-15-00893-s001.zip › nutrients-2181718-supplementary.pdf]
